# Bone lid technique versus standard technique for treatment of mandibular lesions

**DOI:** 10.1186/s12903-024-04594-y

**Published:** 2024-08-02

**Authors:** Mona S. Sheta, Rehab F. Ghouraba, Marwa T. Ibrahim

**Affiliations:** 1 Oral and Maxillofacial Surgery Department, Faculty of Dentistry, Tanta University, El-Giesh St, Tanta, Gharbia Egypt; 2 Oral Medicine, Periodontology, Oral Diagnosis and Radiology Department, Faculty of Dentistry, Tanta University, El-Giesh St, Tanta, Gharbia Egypt

**Keywords:** Bone lid technique, Piezoelectric surgery, Standard technique, Mandibular lesions

## Abstract

**Background:**

Jaw lesions are frequent in the oral and maxillofacial areas. Different methods for enucleating jaw lesions in the oral and maxillofacial sites have been proposed, including the bone lid technique.

**Purpose:**

The aim of this study was to compare the clinical and radiographic results of the bone lid technique employing a piezoelectric surgery to the traditional technique in individuals with mandibular lesions.

**Materials and methods:**

A randomized controlled trial was conducted on 24 patients with mandibular lesions. They were randomly allocated into two groups (*n* = 12 for each group). Group I: the mandibular lesion was excised with bone lid technique using a piezoelectric device, followed by the fixation of the bony window after its repositioning. Group II: the lesion was excised with the traditional method using rotatory burs. Pain, soft tissue healing, bone exposure, bone lid integration, and the volume of the residual bone defect were all assessed clinically and radiographically after one week, one month, and six months.

**Results:**

All patients in both groups showed adequate soft tissue healing except for one case in group I experienced wound dehiscence and bone lid exposure. The bone lid group reported significantly less pain than the usual approach at the 3rd and 7th days. After six months, the volume of bone defect filling was considerably higher in the bone lid group compared to the conventional group.

**Conclusion:**

The bone lid technique was an effective procedure in the management of mandibular lesions compared to the standard method. Besides, this technique provides better bone healing and reduces bone loss.

**Trial registration:**

This clinical trial was registered at clinicaltrials.gov on 14/8/2023 and had registration number NCT05987930.

## Introduction

Jaw lesions are prevalent in the oral and maxillofacial regions. Cystic jaw lesions and impacted teeth are two of the most prevalent situations requiring surgical intervention by oral and maxillofacial surgeons [1, 2].

Surgeons commonly utilize a traditional technique to treat lower jaw lesions, which includes removing the buccal bone plate to give visual and surgical access [3]. The removal of the buccal bone plate can result in volumetric bone defects because ostectomies are performed sufficiently to make the disease accessible and clearly visible during surgery [4]. Furthermore, the mandibular canal and the presence of vital teeth may make surgical access to the lesion problematic [3].

Various alternative treatments or procedures to the treatment of large cystic lesions have been offered, including the bone lid procedure. Bone lid surgery procedures in the maxillofacial region consist of osteotomy of the bony window and complete elevating of the cortical bone. After the procedure, this temporarily removed piece of the cortical bone is returned to its initial location [5]. Besides, using piezoelectric surgery enables precision cutting of the bone lid, tiny osteotomy margins, which reduce bone loss and allow lid repositioning [6].

The bone lid technique aims to reduce the creation of substantial bone defects, improve intraoperative vision, provide additional support for the mucoperiosteal flap, and promote bone formation after healing [3, 6]. According to the previous data, this study was aimed to evaluate the clinical and radiographic outcomes of the bone lid technique with piezoelectric surgery to the standard procedure in patients with mandibular lesions. Our aim in this study was to test the null hypothesis, which states that there is no difference in the volume of bone defect filling across the groups.

## Material & methods

This prospective randomized controlled study followed CONSORT reporting guidelines [7] and included 24 patients with mandibular lesions, who were examined clinically and radiographically using cone beam computed tomography (CBCT) at the Oral and Maxillofacial Surgery Department, Faculty of Dentistry, Tanta University, between June 2023 to February 2024.

The research for this study received approval from Tanta University’s Faculty of Dentistry Research Ethics Committee under code (#R-OS-6-23-7). The patients’ objectives for participating in the study were described to them in accordance with the standards for human research approved by the Research Ethics Committee of the Faculty of Dentistry at Tanta University, which follows the ethical guidelines outlined in the 1964 Helsinki Declaration and its subsequent revisions, and the patients or their parents were given informed consent prior to beginning treatment. NCT05987930 is the approved clinical trial number.

### Eligibility criteria

**Fig. 1:**
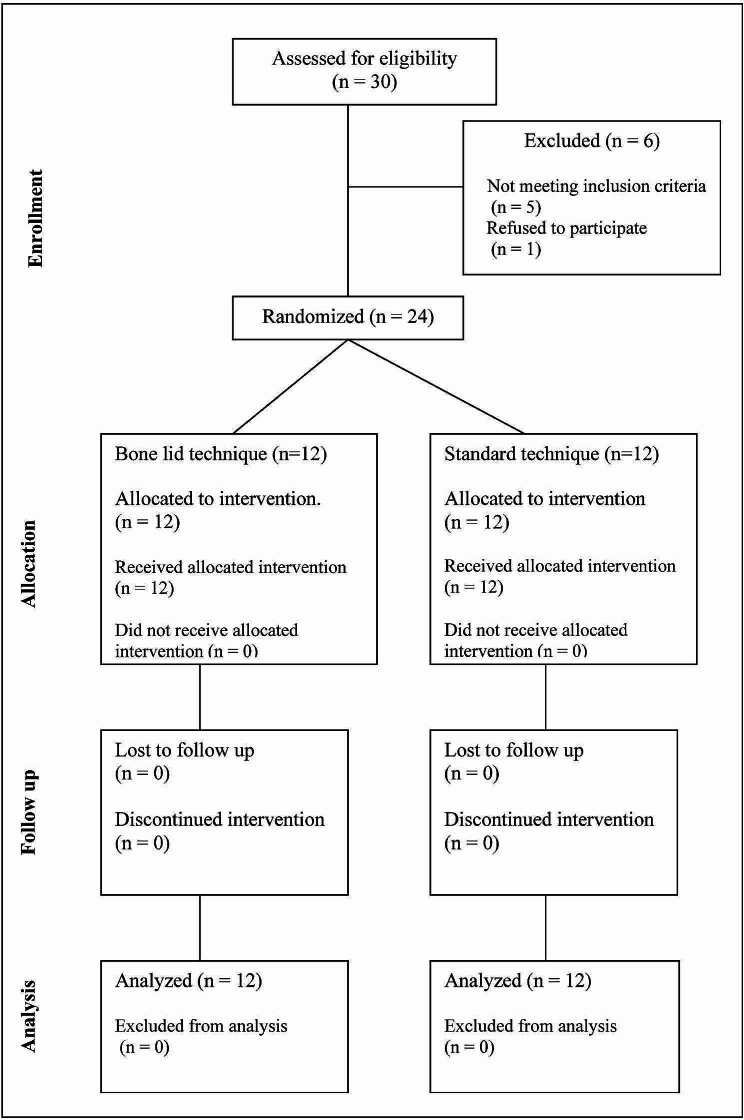
CONSORT flow chart

Patients were selected according to the following main inclusion and exclusion criteria. The main inclusion criteria were patients complaining from the presence of mandibular bony lesion with diameter more than 1 cm and a normal residual buccal cortical plate with a thickness of ≥ 1 mm are existed to allow repositioning and fixation. The exclusion criteria were patients with systemic disease that could interfere with healing, medications that could interfere with bone formation (antiangiogenic medications or anti-resorptive agents) and patients who had received radiotherapy to the head and neck, (Fig. 1).

### Sample size calculation, randomization, and blinding

Using the G*Power Version 3.1.9.4 computer program, the sample size was calculated. This power analysis used the volume of bone defect filling as the primary outcome. The effect size d = 1.787 was calculated based on the results of Abu Hawa et al. [8]. The study’s power had been set at 80%, and the alpha (α) level was (5%) and the beta (β) level was (20%). The estimated sample size was 10 patients for each group. The sample size will be increased to 12 patients per group (total sample size = 24) to compensate for a dropout rate of about 20%.

Twenty-four patients were randomly assigned to two groups via sealed envelopes with an allocation ratio of 1:1 and divided into:

#### Group I

Twelve patients (*n* = 12) in whom the lesion was accessed with the bone lid technique using piezoelectric surgery.

#### Group II

Twelve patients (*n* = 12) in whom the lesion was accessed with standard technique using rotatory bur.

Blinding was limited to the investigator responsible for data analysis.

### Preoperative evaluation

The patient’s history includes personal information (name, age, and gender) were recorded and all patients in both groups underwent:

#### Clinical examination

facial asymmetry, swelling, infection, pain, mobility and vitality of teeth related to the lesion, bone expansion and aspiration biopsy.

#### Radiographic examination

using (CBCT) to evaluate the extension and the volume of lesion, the thickness of the cortical plate or the encroachment of the lesion to the inferior alveolar nerve (IAN).

### Surgical procedure

**Fig. 2:**
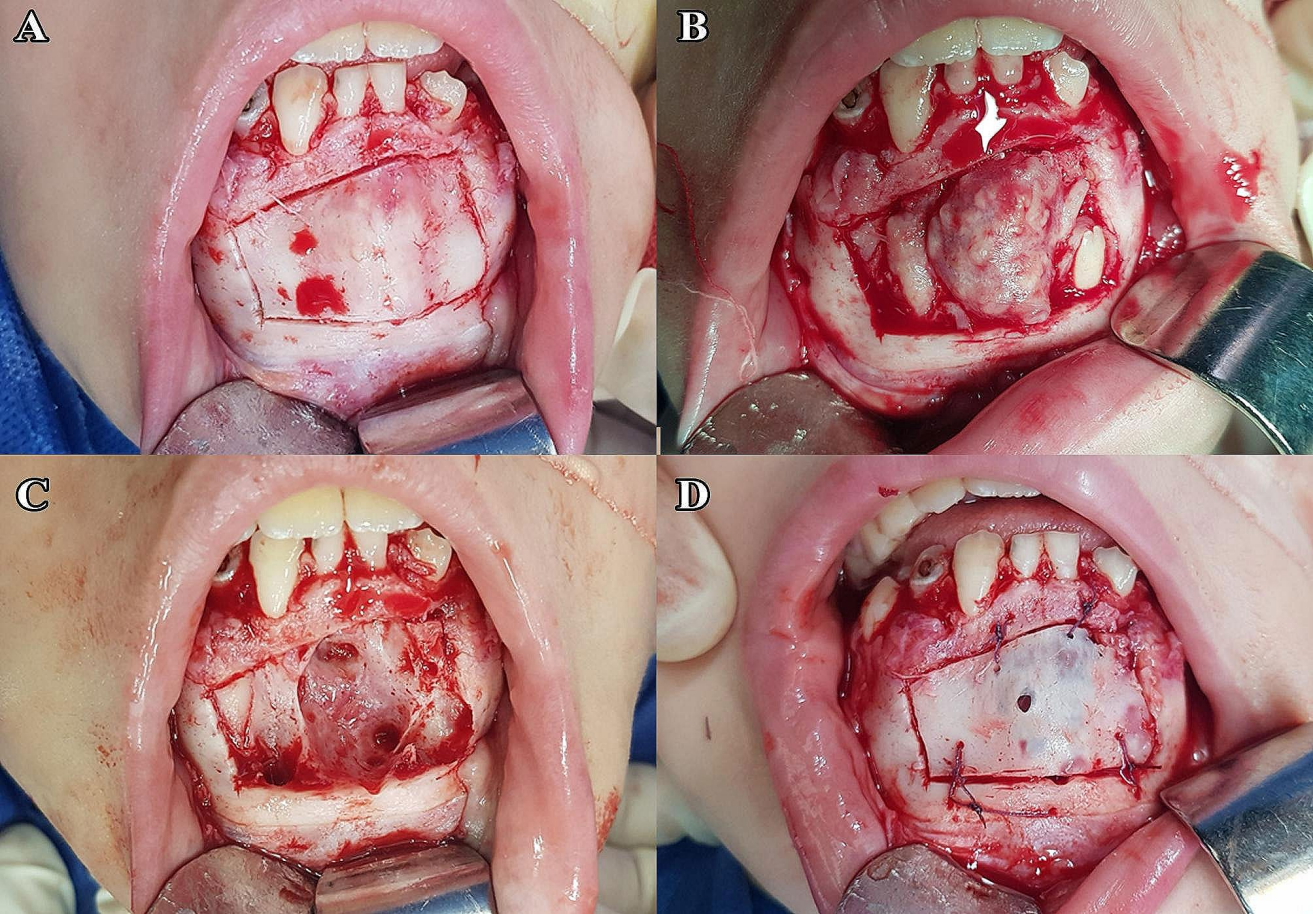
(**A**) Reflection of mucoperiosteal flap and osteotomy of bony window using piezoelectric device (**B**) Removal of the bone lid, providing access to the lesion, (**C**) Excision of the lesion, (**D**) Repositioning of the bone lid and its fixation with 2/0 vicryl, case No. 3, group (I)

**Fig. 3:**
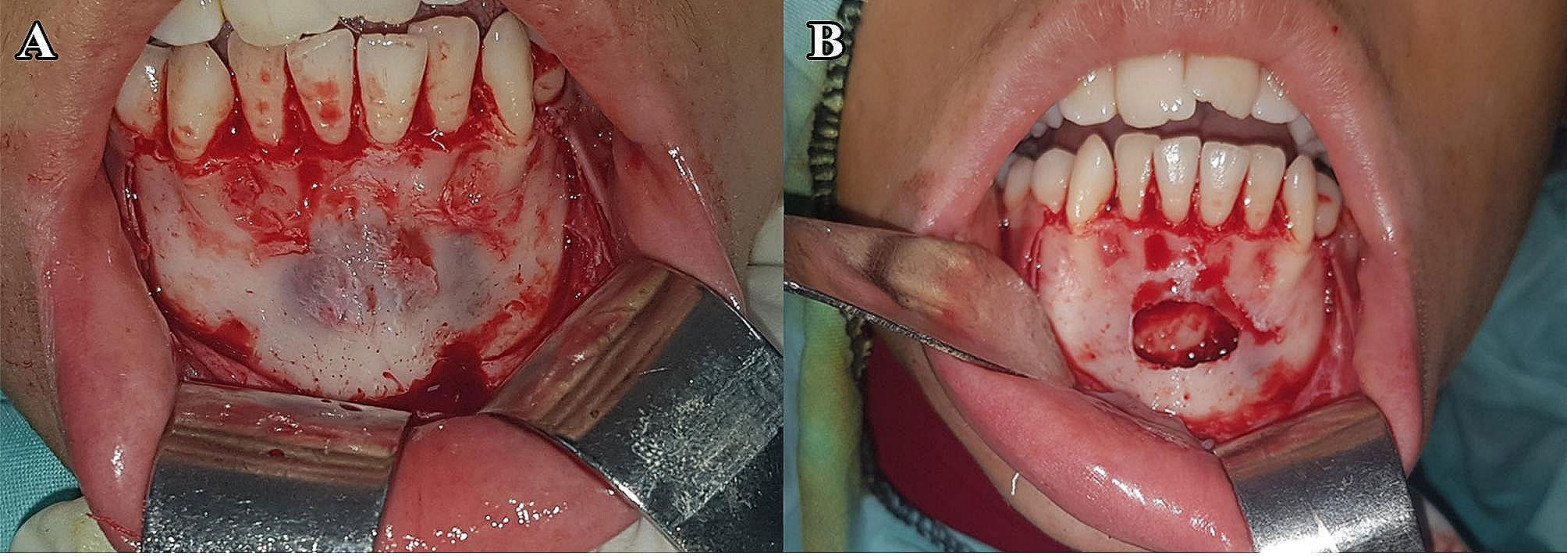
(**A**) Reflection of mucoperiosteal flap showing the monocortical expansion of the buccal plate of bone which was removed with rotatory surgical bur, (**B**) Enucleation of the cyst, case No. 2, group (II)

All procedures were operated under local anesthesia, with the exception of five individuals that required general anesthesia. A full thickness mucoperiosteal flap of adequate size was elevated to gain access to the underlying bone. According to the extension of the lesion in the radiograph, the amount of buccal bone either removed or osteotomized was determined. In group I (bone lid technique), the bony window borders were osteotomized 3–5 mm beyond the predetermined radiographic extension of the lesion using the piezoelectric device and the tip was directed through the cortical bone to underlying cancellous bone with a beveled orientation to facilitate repositioning. A chisel was used gently to free the bony window to avoid the fracture of bony lid and it was placed in sterile saline wetted gauze until enucleation of the lesion. After that the bone lid was replaced in its original position and was fixed using 2/0 sterile absorbable vicryl sutures (Fig. 2). In group II (standard technique) the buccal bone was removed with rotatory surgical bur to get access to the lesion to be enucleated (Fig. 3). The lesion was sent for pathological examination.

The mucoperiosteal flap was repositioned in its original position and primary wound closing was achieved using 3/0 sterile absorbable vicryl sutures.

### Postoperative care

The postoperative care was consistent for all patients. Following surgery, the patients were given antibiotics, nonsteroidal anti-inflammatory medications, and a mouth rinse containing 0.12% chlorhexidine Di-gluconate twice daily for 7 days. The stitches were removed after a week.

### Postoperative evaluation

#### Clinical evaluation

All patients in both groups were assessed at one week, one month, and six months later. The post-surgical pain was assessed using a visual analog scale (VAS), with 0 indicating no pain and 10 indicating the most severe pain. Patients were asked to score the pain based on severity on the first, third, and seventh days. Soft tissue healing was evaluated at one week, two weeks then one month after surgery using the wound healing index (WHI) which scored soft tissue healing according to the following criteria: score 1 = uneventful healing with no gingival edema, erythema, suppuration, patient discomfort, or flap dehiscence; score 2 = uneventful healing with slight gingival edema, erythema, patient discomfort, or flap dehiscence, but no suppuration; and score 3 = poor wound healing with significant gingival edema, erythema, patient discomfort, flap dehiscence, or any suppuration [9].

#### Radiographic evaluation

All patient were exposed to CBCT scan prior lesion removal and 6 months post-surgical using CBCT machine (KaVo OP 3D Vision, Kavo Dental, Biberach, Germany) with fixed exposure parameters (120 Kv, 5 mA and 0.125 mm voxel size) with using the field of view (8D, 8Hcm). On Demand software regarding its 3D module was used version 1.0 (build 1.0.10.7462), × 64 Edition, copyright 2004–2017 Cybermed, Korea and license key 670,094,709) to measure the volume of the lesion using pick tool by calculating the density of the lesion first which differs from surrounding bone then upon the selected density, the volume of lesion was calculated prior to the surgery and after six months to measure the residual bone defect volume and calculate the bone defect filling volume Besides the evaluation of the integrity of the buccal bone lid and any signs of recurrence of the lesion.

### Statistical analysis

Data was collected and tabulated. Descriptive analysis was applied for numerical data, the Mann-Whitney test was used for inter-group comparison with being *P*-value ≤ 0.05 was considered a significant difference (*) and Wilcoxon test for intra-group comparison while chi-square test was used in case of nominal data for inter-and inter group comparison with being *P*-value ≤ 0.05 was considered a significant difference (*).

## Results

Twenty four patients with mandibular lesion were included and randomly divided into two groups in this study. Group I (bone lid technique), twelve patients (8 male and 4 female) had a mean age of 18.5 years (range from 5 to 36 years), ten patients had cystic lesions and the other two had an odontoma. Group II (standard technique), twelve patients (7 male and 5 female) had a mean age of 19 years (range from 7 to 32 years), eleven patients had cystic lesions and one had an odontoma, (Table 1).

**Table 1:** Demographic data and lesion features

Group I					Group II				
No	age	gender	site	Pathology type	No	age	gender	site	Pathology type
1	18	female	posterior	Residual cyst	1	14	female	posterior	Radicular cyst
2	5	male	posterior	Dentigerous cyst	2	12	female	anterior	Primary bone cyst
3	9	male	anterior	odontome	3	29	male	posterior	Radicular cyst
4	23	female	posterior	Radicular cyst	4	32	female	anterior	Infected cyst
5	13	female	posterior	Dentigerous cyst	5	16	female	anterior	odontome
6	7	male	anterior	odontome	6	24	male	posterior	Infected cyst
7	34	male	posterior	Radicular cyst	7	8	male	posterior	Dentigerous cyst
8	21	male	posterior	Residual cyst	8	31	female	posterior	Residual cyst
9	16	female	anterior	Infected cyst	9	10	male	posterior	Calcifying odontogenic cyst
10	8	male	posterior	Dentigerous cyst	10	19	male	anterior	Radicular cyst
11	36	male	posterior	Radicular cyst	11	7	male	posterior	Dentigerous cyst
12	19	male	posterior	Radicular cyst	12	26	male	posterior	Residual cyst

### Clinical results

The primary wound healing was observed in all cases of both groups at one week postoperative and assessed using (WHI) which scored 1 in all cases of group I, except case No. 6 scored 2 and treated with local debridement and oral hygiene measures (daily irrigation) until healing was achieved by secondary intension at the end of the first month. While group II, (WHI) was 1 for all cases. One month postoperatively, WHI was 1 for all cases in both groups.

The mean postoperative VAS pain level in group I was 6.26, 3.22, 1.26 at the first- day, the third and the seventh day postoperative follow-up day respectively and there was significant decrease in pain level between follow-up periods. In group II, the mean postoperative VAS pain level was 6.52, 3.39, 1.42 at the first- day, the third and the seventh day postoperative follow-up days respectively in which there was no significant decrease in pain degree between the first and third day but significantly decrease in the seventh day. There was no significant difference between the groups on the first day. However, on the third and seventh day the pain in bone lid group was significantly less than the standard group, (Table 2).

**Table 2:** Intra-group and inter-group comparison of the visual analog scale results

	Group 1	Group 2	X^2^	*p*₪
Pain at first day post-surgical:				
Mean	6.26	**6.52**	**2.058**	**0.0569ns**
Pain at third day post-surgical:				
Mean	3.22	**3.39**	**3.36**	0.0008***
Pain at seventh day post-surgical				
Mean	1.26	**1.42**	**6.23**	< 0.0001***
*X* ^*2*^	**5.82**	**3.91**		
p^∞^	0.0001***#	0.0264*##		
	# (group I) All follow up periods were a statistically significant from each other ## (group II) There were a statistically significant between (first day, seventh day) and (third day, seventh day)			

*p*₪ Mann−Whitney, p∞ Wilcoxon test: test significance: p*<0.05, p**<0.01, p***<0.001; ns=not significance

### Radiographic results

**Fig. 4:**
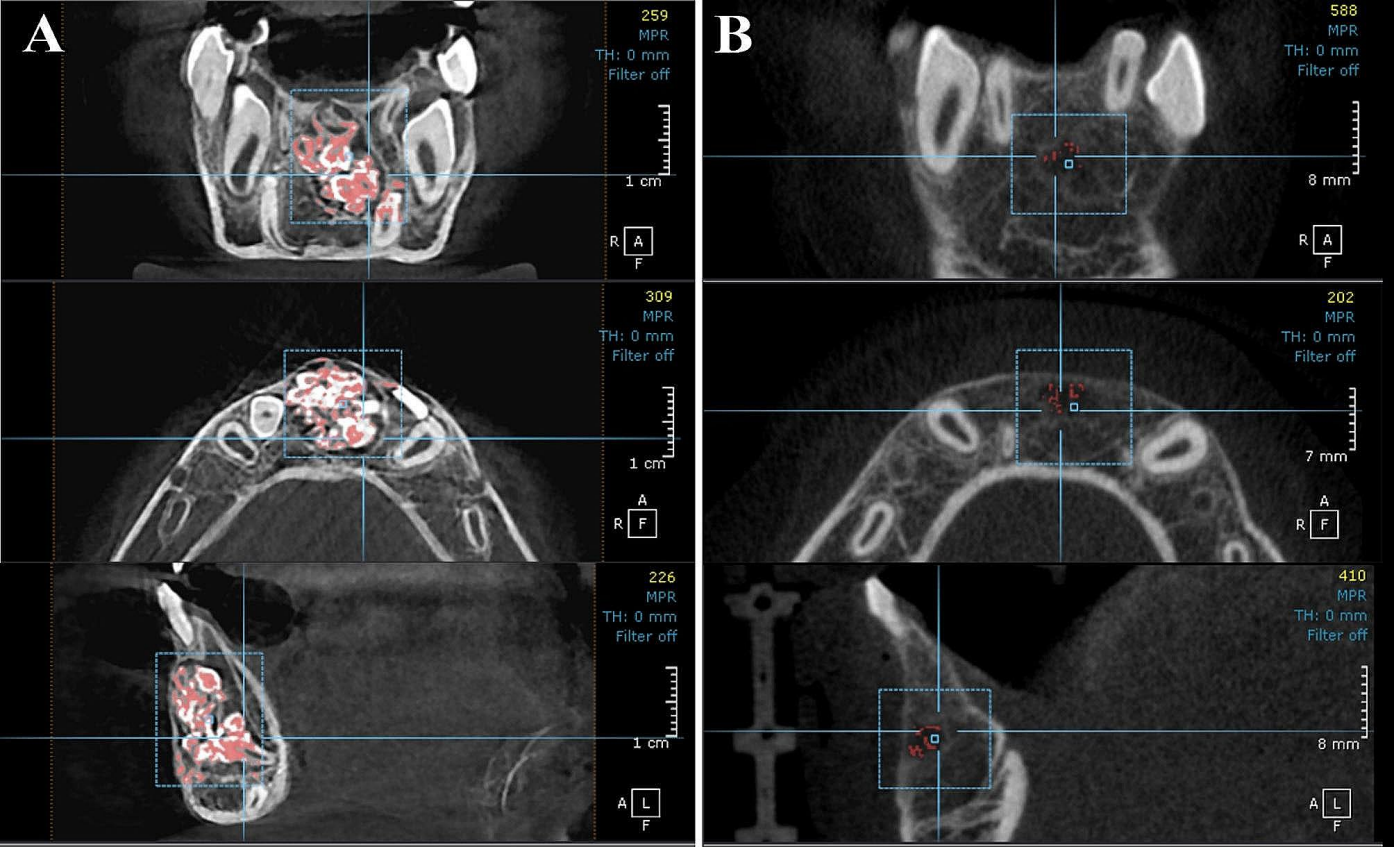
(**A**) Preoperative CBCT showing the volume of lesion, (**B**) Postoperative CBCT showing minimal residual bone defect with integration of buccal cortex, case No. 3, group (I)

**Fig. 5:**
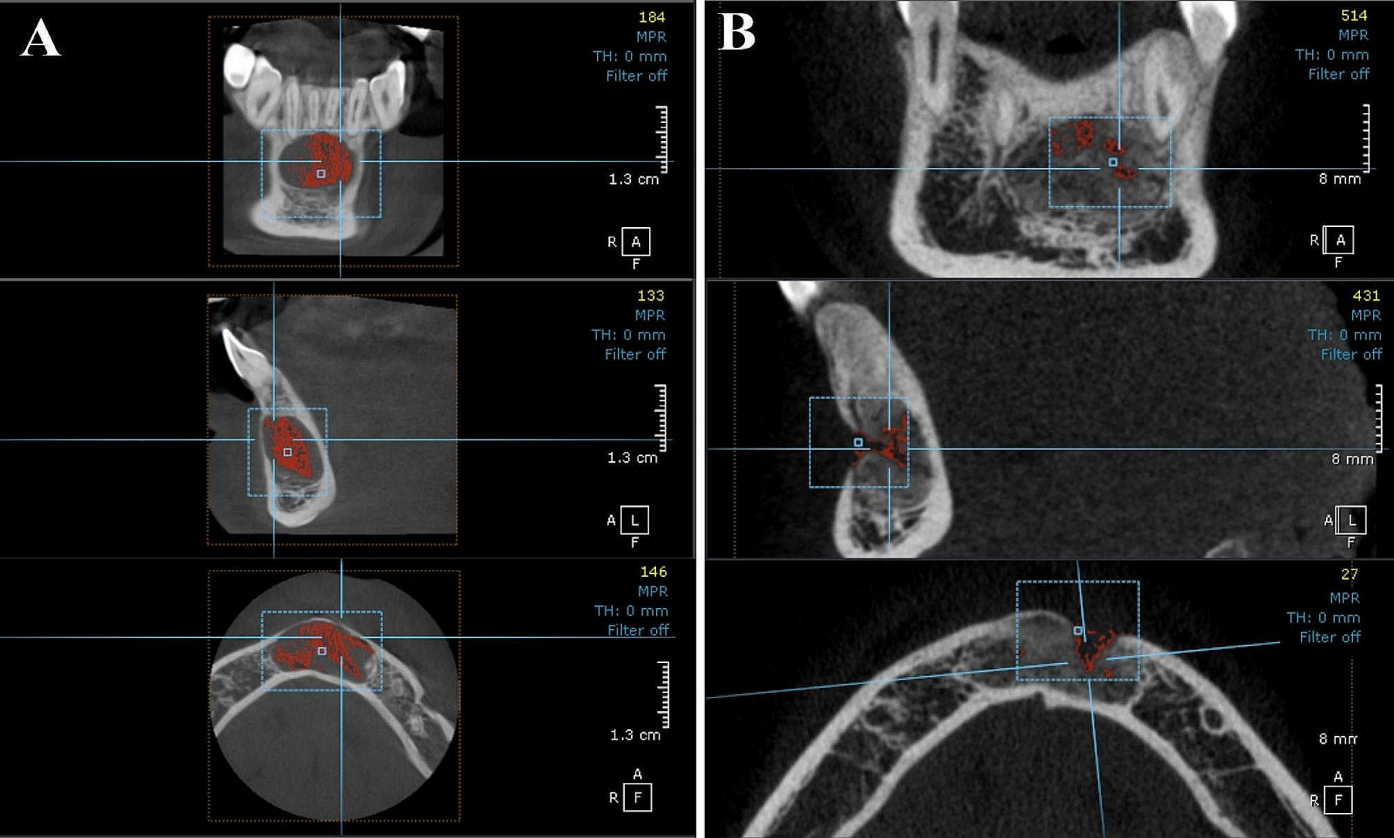
(**A**) Preoperative CBCT showing the volume of lesion defect, (**B**) Postoperative CBCT showing the residual bone defect, case No. 2, group (II)

Bone lid healing, recurrence of the lesion, and the volume of bone defect filling were assessed six months post-surgery using CBCT. There was no radiographic evidence of recurrence or soft tissue invasion within the bone defect in both groups. In group I, the bone lid integration was adequate with surrounding bone except case No. 6 in which the bone lid was exposed and trimmed. There was no significant difference in the preoperative volume of the lesion between both groups as *p* value ≥ 0.05. Six months later, the mean of postoperative bone defect filling volume in group I (bone lid group) was 638.8 ± 112.5 mm^3^, while the mean of group II was 469.7 ± 166.9 mm^3^ which was significantly higher in group I when compared to group II as a result of that the volume of the residual bone defect was significantly less in group I in comparison to group II as *p* value ≤ 0.05 as shown in Figs. 4 and 5 and Table 3.

**Table 3:** Intra-group and inter-group comparison of the mean values of bone volumes

	Group 1	Group 2	*p*₪
Volume at baseline:			
M ± SD	645.0 ± 116.6	515.8 ± 175.1	0.0606ns
Median	638.7	561.3	
Volume of residual defect post-surgical:			
M ± SD	6.259 ± 6.215	46.10 ± 20.40	< 0.0001***
Median	6.959	40.90	
Volume of bone filling:			
M ± SD	638.8 ± 112.5	469.7 ± 166.9	0.0141*
Median	631.7	501.5	
Z	78.00	78.00	
p^∞^	0.0005***	0.0005***	

*p*₪ Mann−Whitney, p∞ Wilcoxon test: test significance: p*<0.05, p**<0.01, p***<0.001; ns=not significance

## Discussion

The bone lid approach is utilized for a variety of procedures, including highly impacted tooth extractions and dental or alveolar bone lesions. The bone lid approach is utilized to access lesions deep within the jawbone. The standard technique utilized for deep jaw bony lesions is usually quite invasive since it requires the removal of massive bones, resulting in the loss of the jaw bone [6]. Thus, the bone lid method, which combines a reliable surgical field of vision with jawbone preservation, is of great significance to oral and maxillofacial surgeons [10].

The purpose of this study was to compare the clinical and radiographic outcomes of the bone lid technique employing a piezosurgery device to the standard method of excision of mandibular lesions using rotatory burs. The use of the bone lid technique in this study allowed for the entire removal of jaw bony lesions and accurate evaluation of recurrent surgical fields with no risk of recurrence, which was consistent with the findings of other maxillofacial surgeons [10].

The piezoelectric device utilized in this study allowed for more precise and fine osteotomies, resulting in less lost bone and easier return of the bone lid to its initial position. This is consistent with Schlee et al. [11] who reported that the piezoelectric device allows for precise placement of the cut on the bony surface, whereas conventional bone drilling or sawing increases the risk of soft tissue damage due to the rotational and torsional force needed to remove the dense, calcified tissue.

In the current study, piezosurgery also results in a clean operative field with minimum bleeding. Schlee et al. [11] indicated that the cavitation effect caused by the cooling fluid supply and the sort of vibration the instrument produced effectively washes away the blood, resulting in optimal visibility in the operative field.

The results of this study revealed that there was no significant difference in the mean VAS pain level between both groups on the first postoperative day. While the mean VAS pain level was significantly decreased in the bone lid group in comparison to the standard technique on the third and seventh postoperative days (*p* value ≤ 0.05), These findings agreed with the results of other studies that reported the employing a piezoelectric device for the enucleation of mandibular cysts lowers postoperative discomfort and edema [12–14].

In this study, the bone lid was fixed using an absorbable suture, which was sufficient to achieve stability. Additionally, the bone lid was cut with an internal beveled edge, which allows for easier repositioning of the lid in its original position and a higher degree of stability. The fixation offers mechanical support, reduces micro motion, and allows the bone lid to conform more closely to the surrounding bone, resulting in better integration. This is consistent with the recommendations of Uchida et al. [15].

The bone lid showed adequate integration in this study except in case No. 6, group I, who suffered from exposure to the bone and failure of its integration as the osteotomy of the bone extended to the alveolar crest, which put it at greater risk of failure. This is based on previous research by Sukegawa et al. [16], who reported that the closer the distance of the lesion to the alveolar crest, the greater the danger of necrosis as the surgical site is closer to the oral side. The recorded complications in this study matched those in other studies conducted by Sivolella et al. [17] and Abu Hawa et al. [8]. The most documented complication was bone exposure and its necrosis.

Oh et al. [18] reported a higher complication rate with bone lids than with conventional ostectomy to access mandibular cysts. This is probably because they used the bone lid technique for extensive large cysts which increased the probability of complications Furthermore, the periosteum is destroyed when the lesion enlarges and comes into contact with the oral mucosa. As a result, it is believed that the risk of dehiscence due to periosteal abnormalities is also connected with poor wound closure in the oral mucosa following surgery [19]. So the size of the lesion, its aggressiveness besides the thickness of the bone lid are fundamental factors should be taken in consideration while planning for surgery.

Six months later, the volume of bone defect filling in the bone lid group was considerably greater than that in the conventional group. This was consistent with Abu Hawa et al. [8], who found that bone defect filling, was 62.7% in the traditional group and 87.1% in the bone lid group following a six-month follow-up. Sivolella et al. [17] investigated bone healing in 11 patients treated with the bone lid approach and found a rate of 93.8% of bone defect was healed after 12 months. Liu et al. [20] used a digital template in the bone lid technique during enucleation of large mandibular cysts and radiographic examination revealed that osteogenesis occurred in the bone cyst defect area in all six cases, with no signs of bone resorption, or cyst recurrence. Younes et al. [21] and Abdelazez et al. [22] also reported that the using of bone lid technique for enucleation of mandibular cysts was valid and safe.

The maintaining of the buccal bone in bone lid technique appeared to have contributed to the adequate bone formation into the defect as it offers an isolated space, allowing the osteogenic cell population originating from the internal walls of the defect to easily repopulate the osseous wound while preventing the non-osteogenic cell population, primarily the epithelium, fibroblast, and other connective tissue cells, from invading the cavity. It follows the biological concepts and essential principles of directed bone regeneration [23, 24], which explains why the bone lid approach outperformed the standard technique in terms of bone defect filling in our investigation.

## Conclusion

This study demonstrated that the bone lid treatment conducted using a piezoelectric device was successful and safe for managing mandibular lesions. It also minimizes bone loss and greatly increases the volume of bone defect filling when compared to the traditional technique employing rotatory burs.

## Data Availability

The data of the submitted paper are available upon request to the corresponding author, mona_sheta@dent.tanta.edu.eg.

## References

[1] Fomete B, Osunde OD, Ogbeifun J, Agbara R, Ononiwu CN. A 10-Year retrospective analysis of 64 cases of cystic lesions of the oral and Maxillofacial Region in a Nigerian Tertiary Hospital. Oman Med J. 2016;31(6):434–8. 27974959 10.5001/omj.2016.87 PMC5099396

[2] Dalmao O, Dempster L, Caminiti MF, Blanas N, Lam DK. Public and professional perceptions of the scope of practice of oral and maxillofacial surgeons. J Oral Maxillofac Surg. 2021;79:18–35. 33386084 10.1016/j.joms.2020.08.009

[3] Chen KW, Chang WC, Wu CT, Hsu PC, Gao HW, Chen YW. New technique for Prevention of the Wound Dehiscence after Jaw Bone Surgery-A Case Report. J Taiwan Oral Maxillofacial Surg Soc. 2015;26:84–92.

[4] Khoury F. The bony lid approach in preimplant and implant surgery: a prospective study. Eur J Oral Implantol. 2013;6:375–84. 24570982

[5] Khoury F, Hensher R. The bony lid approach for the apical root resection of lower molars. Int J Oral Maxillofac Surg. 1987;16(2):166–70. 3110314 10.1016/S0901-5027(87)80125-X

[6] Sivolella S, Brunello G, Berengo M, De Biagi M, Bacci C. Rehabilitation with implants after bone lid surgery in the posterior mandible. J Oral Maxillofac Surg. 2015;73(8):1485–92. 25896563 10.1016/j.joms.2015.03.050

[7] Schulz KF, Altman DG, Moher D. CONSORT 2010 statement: updated guidelines for reporting parallel group randomised trials. J Pharmacol Pharmacotherapeutics. 2010;1(2):100–7. 10.4103/0976-500X.72352 PMC3043330 21350618

[8] Abu Hawa MH, Shehri Z, Alkhouri I. Comparison between the bone lid technique and the traditional technique in Surgical Treatment of the posterior mandibular lesions: a Randomized Controlled Trial. Cureus. 2022;14(6):e26223. 35911276 10.7759/cureus.26223 PMC9312524

[9] Huang LH, Neiva RE, Wang HL. Factors affecting the outcomes of coronally advanced flap root coverage procedure. J Periodontol. 2005;76(10):1729–34. 16253095 10.1902/jop.2005.76.10.1729

[10] Xu GZ, Yang C, Fan XD, Hu YK, Yu CQ. Functional surgery for the treatment of dentigerous cyst in the maxillary sinus. J Craniofac Surg. 2015;26(2):e84–6. 25723658 10.1097/SCS.0000000000001287

[11] Schlee M, Steigmann M, Bratu E, Garg AK. Piezosurgery: basics and possibilities. Implant Dent. 2006;15(4):334–40. 17172949 10.1097/01.id.0000247859.86693.ef

[12] Arakji H, Shokry M, Aboelsaad N. Comparison of piezosurgery and conventional rotary instruments for removal of impacted mandibular third molars: a randomized controlled clinical and radiographic trial. International journal of dentistry. 2016;2016. 10.1155/2016/8169356 PMC5002292 27597866

[13] Mantovani E, Arduino PG, Schierano G, et al. A split-mouth randomized clinical trial to evaluate the performance of piezosurgery compared with traditional technique in lower wisdom tooth removal. J Oral Maxillofac Surg. 2014;72:1890–7. 25234524 10.1016/j.joms.2014.05.002

[14] Pappalardo S, Guarnieri R. Randomized clinical study comparing piezosurgery and conventional rotatory surgery in mandibular cyst enucleation. J Craniomaxillofac Surg. 2014;42:e80–5. 23932541 10.1016/j.jcms.2013.06.013

[15] Uchida T, Yoshida T, Kashiwagi K, Lee S, Kobayashi W, Takahashi K, Murai M, Sato S, Ito K. Clinical, radiographic, and histologic evaluation of localized ridge augmentation using a mandibular bone block. Int J Periodontics Restor Dent. 2008; 28(2). 18546814

[16] Sukegawa S, Yamamoto N, Matsuyama T, Takabatake K, Kawai H, Nagatsuka H, Furuki Y. Factors of successful treatment using the bone lid technique in maxillofacial surgery: a pilot study. J Hard Tissue Biol. 2021;30(2):193–8. 10.2485/jhtb.30.193

[17] Sivolella S, Brunello G, Fistarol F, Stellini E, Bacci C. The bone lid technique in oral surgery: a case series study. Int J Oral Maxillofac Surg. 2017;46(11):1490–6. 28716472 10.1016/j.ijom.2017.06.027

[18] Oh S, Park JH, Paeng JY, Kim CS, Hong J. Comparison of surgical approach and outcome for the treatment of cystic lesion on lower jaw. J Korean Association Oral Maxillofacial Surg. 2012;38(5):276–83. 10.5125/jkaoms.2012.38.5.276

[19] Consolaro A. Dehiscences and fenestrations: methodological care necessary to avoid errors in diagnosis and measurement. Dent Press J Orthod. 2017;22(5):25–9. 10.1590/2177-6709.22.5.025-029.oin PMC5730133 29160341

[20] Liu Z, Huang D, Li K, Li H, Liu L. Precise locating and cutting of the bone lid with a digital template during the treatment of large mandibular cysts: a case series study. J Cranio-Maxillofacial Surg. 2021;49(5):358–61. 10.1016/j.jcms.2021.01.010 33581955

[21] Younes R, Nasseh I, Lahoud P, Wassef E, Dagher M. Bone lid technique using a piezoelectric device for the treatment of a mandibular bony lesion. Case Rep Dentistry. 2017;2017(1):9315070. 10.1155/2017/9315070 PMC5738583 29362679

[22] Abdelazez AK, Hany HE, El Din ME, El Meregy MM, Abdelhameed AM, El-Kabany IM, Abdelraouf AM, Salah M, El Hadidi YN, El Abdien MD. The evaluation of the effect of performing guided lid surgery with enucleation of a cystic lesion; a case report. Int J Surg Case Rep. 2022;97:107385. 35868132 10.1016/j.ijscr.2022.107385 PMC9403024

[23] Dahlin C, Linde A, Gottlow J, Nyman S. Healing of bone defects by guided tissue regeneration. Plast Reconstr Surg. 1988;81(5):672–6. 3362985 10.1097/00006534-198805000-00004

[24] Hämmerle CH, Schmid J, Lang NP, Olah AJ. Temporal dynamics of healing in rabbit cranial defects using guided bone regeneration. J Oral Maxillofac Surg. 1995;53(2):167–74. 7830183 10.1016/0278-2391(95)90396-8

